# Efficient CRISPR-rAAV engineering of endogenous genes to study protein function by allele-specific RNAi

**DOI:** 10.1093/nar/gku1403

**Published:** 2015-01-13

**Authors:** Manuel Kaulich, Yeon J. Lee, Peter Lönn, Aaron D. Springer, Bryan R. Meade, Steven F. Dowdy

**Affiliations:** Department of Cellular and Molecular Medicine, University of California, San Diego, 9500 Gilman Drive, La Jolla, CA 92093, USA

## Abstract

Gene knockout strategies, RNAi and rescue experiments are all employed to study mammalian gene function. However, the disadvantages of these approaches include: loss of function adaptation, reduced viability and gene overexpression that rarely matches endogenous levels. Here, we developed an endogenous gene knockdown/rescue strategy that combines RNAi selectivity with a highly efficient CRISPR directed recombinant Adeno-Associated Virus (rAAV) mediated gene targeting approach to introduce allele-specific mutations plus an allele-selective siRNA Sensitive (siSN) site that allows for studying gene mutations while maintaining endogenous expression and regulation of the gene of interest. CRISPR/Cas9 plus rAAV targeted gene-replacement and introduction of allele-specific RNAi sensitivity mutations in the *CDK2* and *CDK1* genes resulted in a >85% site-specific recombination of Neo-resistant clones versus ∼8% for rAAV alone. RNAi knockdown of wild type (WT) Cdk2 with siWT in heterozygotic knockin cells resulted in the mutant Cdk2 phenotype cell cycle arrest, whereas allele specific knockdown of mutant CDK2 with siSN resulted in a wild type phenotype. Together, these observations demonstrate the ability of CRISPR plus rAAV to efficiently recombine a genomic locus and tag it with a selective siRNA sequence that allows for allele-selective phenotypic assays of the gene of interest while it remains expressed and regulated under endogenous control mechanisms.

## INTRODUCTION

**Figure 1.**
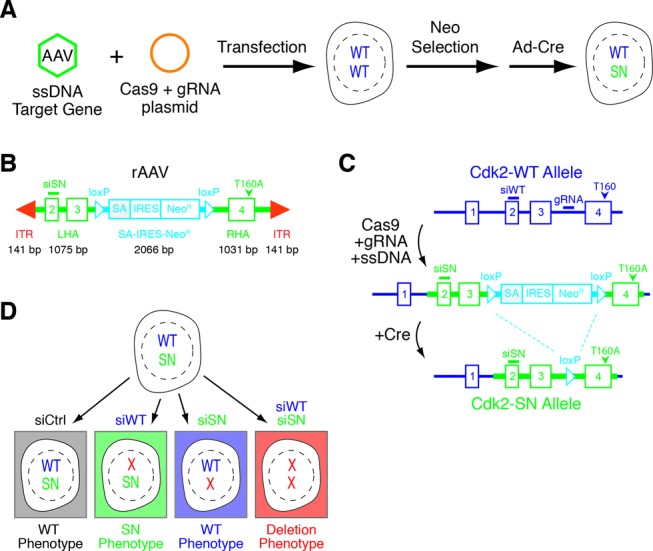
CRISPR/Cas9 plus rAAV targeted recombination. (**A**) Study concept of combining CRISPR/Cas9 plasmid co-expressing a specific guide strand with rAAV. (**B**) Design of rAAV Cdk2 promoter-less construct. (**C**) Graphic depiction of the rAAV-targeting strategy to introduce siSN silent point-mutations into exon 2 and a point mutation at Thr-160 to Ala (T160A) of human Cdk2. Highlighted are the two crucial steps of ‘targeting’ (Cas9 + gRNA + ssDNA) and allele ‘restoration’ (+Cre). Note locations of gRNA, siSN and T160A. (**D**) Introduction of siSN sequence plus a gene mutation into one allele of a given gene allows for selective depletion of the wild type allele by siWT resulting in a mutant phenotype, selective depletion of the mutant gene by siSN resulting in a rescued phenotype or depletion of both alleles to mimic a loss of function phenotype.

The classical way to study mammalian gene function is to genetically knockout the gene or RNAi deplete the mRNA and hence, the protein of interest, to induce a phenotype. To confirm that the targeted gene was the causal gene requires rescuing the loss of function phenotype by ectopic expression of a variant of the same gene. Unfortunately, constitutive gene knockout can activate compensatory mechanisms that significantly impair the phenotype and conclusions to gene function (1,2). In contrast, acute RNAi-mediated gene depletion can reveal additional functions and lead to a more detailed molecular understanding (3). However, rescue of either gene deletion or depletion is equally fraught with potential pitfalls. Commonly used protein expression systems, including stably integrated genomic constructs under inducible promoters (4), often significantly overexpress the rescue protein, which alters the stoichiometry of protein–protein interactions and can lead to potential false-positive ‘rescue’ results (5,6). While the lack of proper gene regulation can potentially be addressed using minigenes or bacterial artificial chromosomes (6), these approaches represent an artificial situation for the cell. To close this gap, we developed a gene knockdown/rescue strategy that works at the endogenous gene level by combining RNAi selectivity with a highly efficient CRISPR–Cas (7–10) directed recombinant Adeno-Associated Virus (rAAV) mediated gene targeting approach to introduce allele-specific phenotypic mutations of interest plus an allele-selective siRNA Sensitive (siSN) site (Figure 1).

## MATERIALS AND METHODS

### CRISPR and rAAV cloning procedures

Cdk2- and Cdk1-specific gRNAs were designed that contain unique sequence between exons 3 and 4 and cloned into pX330 (11). Cdk2 gRNA: 5′-tcattatattcattaaccct-3′ and Cdk1 gRNA: 5′-aatttgtaatttaaggatcg-3′. For in-frame cloning of human Cdk2 genomic locus, DNA oligonucleotides 5′-ttgtacagctcgtccatgccgag-3′ and 5′-tcagagtcgaagatggggtactggc-3′ were used. To generate plasmids refractory to Cdk2 siWT siRNA duplexes, a series of seven silent point-mutations was introduced using the DNA oligonucleotide 5′-atctctctgcttaaggaattgaatcacccgaacattgtcaagctgct-3′ and its corresponding antisense. Left and right homology arms required to target the human Cdk2 locus were generated using genomic DNA of hTERT-RPE1 cells and the following DNA oligonucleotides: LHA-f 5′-ggagaggtgggttgggggccagtagaagg-3′ LHA-r 5′-gcagggaaggagacacaaaaagaagggg-3′, RHA-f 5′-ccctagggttggactgaacaatcaaagttg-3′ and RHA-r 5′-gtttccttccctccatcatctttcccctccc-3′. To introduce siWT refractory mutations into the left homology arm the DNA oligonucleotides 5′-atctctctgcttaaggaattgaatcacccgaacattgtcaagtaagta-3′ and 5′-tacttacttgacaatgttcgggtgattcaattccttaagcagagagat-3′ were used. Left and right homology arms were cloned into pAAV-SEPT (12). Infectious rAAV particles were generated by transfection of 293T cells with 3 μg of donor template containing pAAV-SEPT, and packaging plasmids pAAV-RC and pHELPER. Transfected 293T cells were incubated for 5 days, prior to harvest of cells, followed by four freeze/thaw cycles to release infectious rAAV particles. Cellular debris was separated by centrifugation and rAAV containing supernatant was stored at −80°C.

### Clonal selection of recombined clones

hTERT-RPE1 cells were seeded to a density of 30% (∼2 × 10^5^ cells) in a 10-cm dish and incubated overnight. Cells were washed with phosphate-buffered saline (PBS) and incubated 30 min with 5 ml of serum-free Dulbecco's Modified Eagle (DME)/F12 media (Life Technologies) before 5 ug of ethanol-precipitated gRNA/Cas9-plasmid (pX330) was transfected. After 4 h, cells were washed with fresh DME/F12 media and incubated for an additional 2 h. To transduce cells with rAAV, cells were washed twice with 10 ml Hank's Balanced Salt Solution (HBSS) buffer. Six milliliters of processed rAAV-containing supernatant was mixed with 3 ml of fresh DME/F12 media and added on to cells (13). After 4 h of incubation, an additional 6 ml (15 ml total) of fresh DME/F12 media was added to cells and incubated for 48 h. To start the clonal selection of recombined clones, cells were trypsinized and collected in 20 ml of fresh DME/F12 media supplemented with 400 ug/mL G418. Cells were seeded into 96-well plates, wrapped in plastic to prevent evaporation and incubated for 3 weeks. Wells with multiple colonies were deselected and monoclonal colonies were processed. To remove the inserted floxed SA-IRES-Neo cassette, cells were washed twice with HBSS buffer, given fresh serum-free Dulbecco's modified Eagle's medium (DMEM)/F12 media and transduced with Adenovirus-Cre (University of Iowa) for 12 h, followed by media supplementation with 10% serum.

### PCR-based identification of recombined clones

Cells were grown in a 48-well format, split and 90% of cells were used to extract genomic deoxyribonucleic acid (DNA) with Total Nucleic Acid (TNA) buffer (0.1 M NaCl, 10 mM Tris–HCl pH 8, 1 mM ethylenediaminetetraacetic acid (EDTA) and 10% sodium dodecyl sulfate (SDS)) and phenol:chloroform:isoamyl alcohol 25:24:1. Genomic DNA was precipitated with 3 M Sodium Acetate (NaAc) and 100% Ethanol (EtOH), washed with 75% EtOH and resuspended in water. To identify correct recombined Cdk2 clones, a 10 ul polymerase chain reaction (PCR) reaction with primer pairs P1 and P2 (Supplementary Figure S2A) was performed on Cdk2 using OneTaq^®^ DNA Polymerase (NEB), P1: 5′-atggagaacttccaaaaggtggaaaagatcgg-3′, P2: 5′-ggatccactagttctagagagctcgcaggg-3′. To distinguish between single allele and double Cdk2 allele recombined clones, a second PCR using primers P3 and P4 was performed using primer sequences P3: 5′-ccaagTGAGACTGAGGGTGTGCCCAGTACTGCCATCCG-3′, P4: 5′-ccagagaggataaggaccaagtcttactcaattttccatccc-3′. For Cdk1, the following PCR primers were USED: P1: 5′-GATCTGGAGACAGTCTTTGGGTCAAGGGTAGACAACTCCACAGAAGACAG-3′, P2: 5′-GATCTGGAGACAGTCTTTGGGTCAAGGGTAGACAACTCCACAGAAGACAG-3′, P3: 5′-GGGCTACGCTTAGCTACGTTTGGTAGGTTAGGTGTGTTCAATGC-3′, and P4: 5′-GGCCATTTTAGGCTAATAAGCATTTGTGGGTGGGAAGCCC-3′.

### Allele-specific quantitative real-time PCR

hTERT-RPE1 cells were treated as described above. RNA was extracted using RNeasy kit (Qiagen, Valencia, CA, USA) and reversed transcribed using iSCRIPT cDNA synthesis kit (Invitrogen). cDNAs were assayed on a ABI7300 thermal cycler using the SYBR select PCR master-mix (Applied Biosystems). Each sample was measured in triplicates and the experiments have been repeated at least three times. Relative gene expression normalized to GAPDH was determined using the ddCt algorithm. Primers used were: hCdk2-WT, forward, 5′-GAGCTTAACCATCCTAATATTGTC-3′, reverse, 5′-GCTGGAACAGATAGCTCTTGA-3′; hCdk2-Mut, forward, 5′-GAATTGAATCACCCGAACATTGTC-3′, reverse 5′-GCTGGAACAGATAGCTCTTGA-3′. hGAPDH, forward, 5′-GGAGTCAACGGATTTGGTCGTA-3′, reverse 5′-GGCAACAATATCCACTTTACCA-3′.

### Cell culture and synchronization

Human hTERT-RPE1 cells were cultured at 37°C, in 5% CO_2_ atmosphere in DME/F12 media (Life Technologies), supplemented with 10% heat-inactivated fetal bovine serum (FBS) and penicillin/streptomycin (100 and 100 μg/ml, respectively). Thymidine was obtained from Sigma–Aldrich. For thymidine synchronization, cells were treated with 2 mM thymidine for 24 h prior to cell lysis. For cell cycle exit synchronization, cells were thymidine synchronized and released by PBS wash and subsequently cultured in serum-free DME/F12 media for 60 h. Cell cycle re-entry was obtained by removal of serum-free media and addition of DME/F12 media containing 10% heat-inactivated FBS.

### Cell extracts and immunoprecipitation

Preparation of lysates and immunoblot analyses were performed as described previously (5) using Tris lysis buffer (50 mM Tris–HCl pH 7.8, 150 mM NaCl, 1% IGEPAL CA-630) containing 20 mM NaF, 20 mM β-glycerophosphate, 0.3 mM Na-vanadate, 20 μg/ml RNase A, 20 μg/ml DNase and 1/300 protease inhibitor cocktail (P8340, Sigma–Aldrich) and phosphatase inhibitor cocktail #2 (P5726, Sigma–Aldrich). Antibodies used in this study were purchased from the following sources: rabbit anti-Cdk2 (M2 SC-163, Santa Cruz Biotechnology), mouse anti-actin (ab-3280, Abcam, Cambridge, MA, USA), rabbit anti-GFP (ab-290, Abcam), mouse anti-tubulin (Developmental Studies Hybridoma Bank, University of Iowa), mouse anti-Cyclin A (SC-751, Santa Cruz Biotechnology) and mouse anti-cyclin E (SC-198, Santa Cruz Biotechnology). Secondary antibodies used for western blot analysis were goat anti-mouse (31430) and, goat anti-rabbit (31460, Thermo Scientific). Mouse anti-tubulin hybridoma cell line (clone #12G10) was developed by J. Frankel and E.M. Nelson under the auspices of the NICHD and maintained by the Developmental Studies Hybridoma Bank.

### Fluorescence microscopy

Cells were seeded and grown on coverslips, treated as indicated, and simultaneously fixed and permeabilized for 10 min at RT with 4% PFA and 0.1% Triton-X100 in PBS. Images were acquired using a DeltaVision Olympus IX71 microscope equipped with a ×60/1.42 oil objective. Collection and processing of acquired images was carried out using ImageJ 1.44o (Wayne Rasband, National Institutes of Health, USA).

### Size-exclusion chromatography (SEC)

Asynchronously growing cells were lysed in freshly prepared SEC lysis buffer: 20 mM Tris–HCl, pH 7.4, 150 mM NaCl, 0.5% IGEPAL (CA-630), 1 mM DTT, 20 mM NaF, 20 mM betaglycerophosphate, 20 μg/ml RNase A, 20 μg/ml DNase, 1 μg/ul leupeptin, 1 μg/ul aprotinin, 1/300 protease inhibitor cocktail (P8340, Sigma–Aldrich) and 1/100 phosphatse inhibitor cocktail #2 (P5726, Sigma–Aldrich). One milligram of total protein was loaded onto a Superdex 200 10/300 GL column (GE Healthcare Bio-Sciences) connected to a BioLogic™ Low-Pressure Chromatography Systems (Bio-Rad). A constant flow rate of 0.4 ml/min of an isocratic elution buffer (20 mM Tris–HCL, pH 7.4, 150 mM NaCl) was used and 500 μl fractions were collected after an initial retention volume of 16 ml. Fractions were TCA precipitated and resuspended in 50 μl 2× Laemmli buffer for SDS-page gel electrophoresis and immunoblotting.

### Transient plasmid and siRNA transfection

Plasmid transfection was performed using Lipofectamin 2000 (Life Technologies) according to the manufacturer's instructions. siRNA duplexes were transfected using Lipofectamin RNAiMAX (Life Technologies) according to the manufacturer's instructions. The following siRNA duplexes were synthesized and used: siCTRL passenger 5′-CUUACGCUGAGUACUUCGAUT-3′, siWT passenger 5′-GAGCUUAACCAUCCUAAUATT-3′, and siSN passenger 5′-GAAUUGAAUCACCCGAACATT-3′. siRNA oligonucleotides were used at a final concentration of 25 nM. Plasmid transfections were performed for 48 h with cells synchronized with thymidine. siRNA transfections were done as indicated or performed for 36 h with cells synchronized with thymidine.

### Time-lapse video microscopy

hTERT-RPE1 cells stably expressing histone 2B-GFP were seeded in six-well chambers, treated as indicated and imaged using a Nikon ECLIPSE Ti microscope equipped with a CoolLED pE-1 excitation system and a ×20/0.75 air Plan Apo objective (Nikon). Images were acquired at multiple positions every 20 min. To collect and process data, ImageJ 1.44o software (Wayne Rasband, National Institutes of Health, USA) was used, respectively.

## RESULTS

rAAV mediated gene targeting by homologous recombination is a precise and powerful technique that is widely used to study mammalian gene function (14–16). Although ssDNA rAAV mediated recombination is significantly more efficient than recombination by dsDNA vectors, rAAV mediated gene targeting remains relatively inefficient. To address this problem, we developed a CRISPR directed rAAV gene-targeting strategy that uses the DNA cleavage selectivity of CRISPR/Cas9 to dramatically enhance the rAAV ssDNA mediated recombination frequency while also building in an allele-selective siSN site (Figure 1A). First, we cloned the human Cdk2 genomic region from exons 2 to 4 into the pAAV-SEPT plasmid (Figure 1B) (12). To generate the Cdk2 siSN allele, we introduced seven silent point mutations into exon 2 sequence of Cdk2 (Cdk2-SN) (Supplementary Figure S1A and B). We placed a floxed, in-frame splice acceptor site (SA) and internal ribosome entry site (IRES) cassette followed by a Neomycin resistance (Neo^R^) gene between exons 3 and 4 (Figure 1B). This promoter-less approach requires recombination into the Cdk2 locus to obtain Neo^R^ expression (Figure 1C). To test the specificity of siSN, we co-transfected both wild type (siWT) and siSN siRNAs together with plasmids encoding either GFP-Cdk2^WT^ or GFP-Cdk2^SN^ and analyzed for GFP-Cdk2 expression by immunofluorescence and immunoblotting. Impressively, siWT specifically knocked down GFP-Cdk2^WT^ expression, while siSN restrictively knocked down only the GFP-Cdk2^SN^ construct (Supplementary Figure S1C and D).

**Figure 2.**
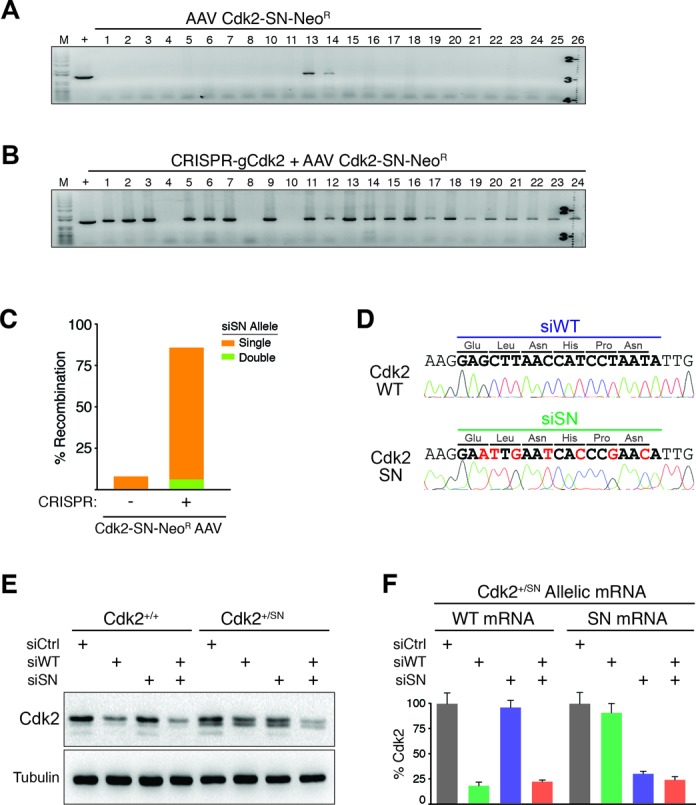
Switching Cdk2 alleles on and off by siRNA. (**A**) Genomic PCR analysis on clones obtained with control Cdk2-SN rAAV infection alone showing 2/26 (<8%) recombination events. (**B**) Genomic PCR analysis on clones obtained with CRISPR-gCdk2 plus Cdk2-SN rAAV with 38/41 (93%) recombination events. (**C**) Bar graph of recombination efficiency of combining CRISPR-mediated DNA-cleavage with rAAV-mediated template delivery analyzed by genomic PCR. (**D**) Allele-specific sequencing results of wild type allele and recombined Cdk2-SN allele. Bold letters indicate siWT or siSN siRNA target sites; red letters indicate seven silent mutations of siSN sequence. (**E**) Cdk2 immunoblot analysis of Cdk2^+/+^ and Cdk2^+/SN^ cells transfected with control siCtrl, siWT, siSN or both, as indicated. (**F**) Bar graph of allele-specific quantitative real-time PCR to detect either WT mRNA or SN mRNA of Cdk2 in RNAi treated Cdk2^+/SN^ cells, as indicated. Data is from three independent experiments; error bars represent standard deviation.

Based on locus and integration specific PCR assays, infection of RPE cells with only Cdk2-SN-Neo^R^ rAAV (no CRIPSR) resulted in 2/26 (<8%) Neo^R^ colonies containing recombined Cdk2-SN single alleles, and no double alleles (Figure 2A) (Supplementary Figure S2A, B and E). In contrast, infection of Cdk2-SN-Neo^R^ rAAV plus transfection of pX330 CRISPR/Cas9 plasmid co-expressing a Cdk2 guide strand RNA (gCdk2) that targets a unique sequence between exons 3 and 4 resulted in an impressive 41/48 (85%) Neo^R^ colonies containing recombined Cdk2-SN (Figure 2B and C) (Supplementary Figure S2D). We confirmed these observations by locus-specific genomic sequencing (Figure 2D). Of the 41 Cdk2-SN colonies, 38 (93%) were single Cdk2-SN alleles (Cdk2^+/SN^) and 3 (7%) were double alleles (Cdk2^SN/SN^) (Supplementary Figure S2G).

Insertion of the SA-IRES-Neo^R^ cassette between exons 3 and 4 of Cdk2 (Cdk2^+/SN-NeoR^) temporarily inactivates the targeted Cdk2 allele and resulted in a 50% reduction of Cdk2 compared to parental Cdk2^+/+^ cells (Supplementary Figure S3A). However, the cell cycle profile of Cdk2^+/SN-NeoR^ clones were indistinguishable from parental cells (Supplementary Figure S3B). Consistent with this observation, size-exclusion chromatography from lysates of asynchronously cycling Cdk2^+/+^ and Cdk2^+/SN-NeoR^ clones showed no significant differences between complexes of Cdk2 with cyclins E and A (Supplementary Figure S3C). Importantly for our subsequent experimentation below, we note that all of the cyclins E and A are bound by Cdk2 in both cell types, demonstrating that 50% reduction of Cdk2 protein has no consequences on efficiency of Cdk2–cyclin complex formation. Treatment of Cdk2^+/SN-NeoR^ clones with Adenovirus-Cre to remove the Neo^R^ cassette restored Cdk2 expression to wild type levels (Figure 2E) (Supplementary Figure S4), demonstrating that the introduced siSN silent point mutations had no detectable consequences on Cdk2 transcription or translation.

To test our initial hypothesis of selectively regulating Cdk2 alleles by RNAi, we transfected either control siCtrl, siWT, siSN or both siWT and siSN into Cdk2^+/+^ and Cdk2^+/SN^ cells. As expected, Cdk2 protein levels were highly reduced when siWT was transfected into Cdk2^+/+^ cells, whereas siSN showed no Cdk2 reduction in Cdk2^+/+^ cells (Figure 2E) (Supplementary Figure S4). Transfection of both siWT and siSN into Cdk2^+/+^ cells was no different from siWT alone. However, transfection of siWT into Cdk2^+/SN^ cells showed only a ∼50% reduction of Cdk2 protein levels (Figure 2E) (Supplementary Figure S4). Likewise, transfection of siSN into Cdk2^+/SN^ cells also resulted in only a ∼50% depletion of Cdk2, whereas transfection of both siSN and siWT resulted in a complete Cdk2 depletion in Cdk2^+/SN^ cells. Cdk2 mRNA allele-specific quantitative RT-PCR revealed that siWT selectively targeted the wild type Cdk2^WT^ mRNA, while siSN only targeted the Cdk2^SN^ mRNA (Figure 2F). These observations demonstrate the ability of CRISPR plus rAAV to efficiently knockin allele selective RNAi sequences into cells.

**Figure 3.**
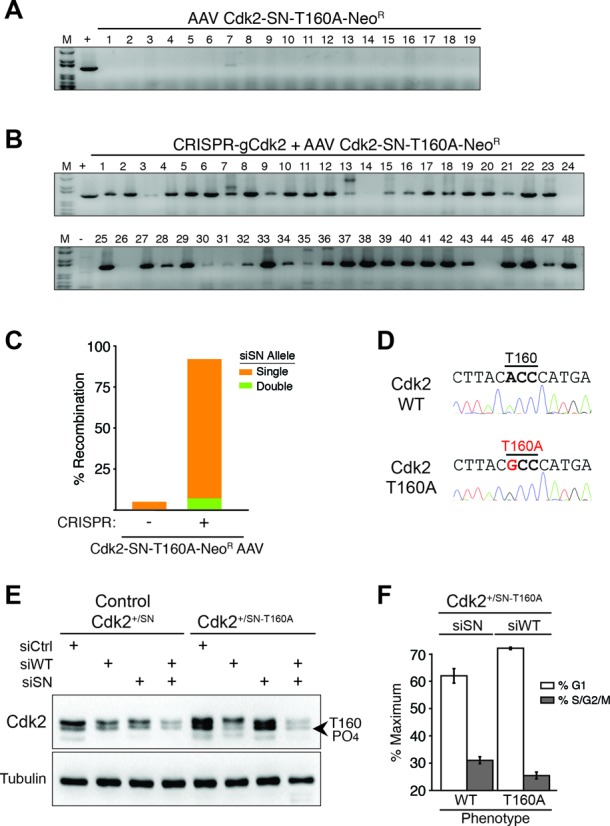
Allele specific RNAi depletion between Cdk2-WT and Cdk2-T160A genotypes. (**A**) Genomic PCR analysis on clones obtained with control Cdk2-SN-T160A rAAV infection alone showing 1/19 (5%) recombination events. (**B**) Genomic PCR analysis on clones obtained with CRISPR-gCdk2 plus Cdk2-SN-T160A rAAV with 44/48 (92%) recombination events. (**C**) Bar graph of recombination efficiency of combining CRISPR-mediated DNA-cleavage with rAAV-mediated template delivery analyzed by genomic PCR. (**D**) Allele-specific sequencing results of wild type allele and recombined Cdk2-T160A allele. (**E**) Cdk2 immunoblot analysis of Cdk2^+/SN^ and Cdk2^+/SN-T160A^ cells transfected with control siCtrl, siWT, siSN or both, as indicated. The location of the T160-PO4 Cdk2 protein is indicated. (**F**) Propidium iodide DNA flow cytometry cell cycle analysis of Cdk2^+/SN-T160A^ cells transfected with either siSN, which depletes Cdk2-T160A and expresses wild type Cdk2 (no phenotype), or siWT, which depletes wild type Cdk2 and expresses Cdk2-T160A, resulting in a cell cycle arrest phenotype. Data is from three independent experiments; error bars represent standard deviation.

To investigate gene function at endogenous gene expression and regulation levels using CRISPR plus rAAV, we introduced an Ala mutation into Cdk2′s activating T-loop phosphorylation site (T160A) in exon 4 and placed the siSN sequence in exon 2 (Figure 1B and C). Based on locus and integration-specific PCR, infection of RPE cells with only Cdk2-SN-T160A-Neo^R^ rAAV (no CRIPSR) resulted in 1/19 (5%) Neo^R^ colonies containing recombined Cdk2-SN-T160A single alleles, and no double alleles (Figure 3A) (Supplementary Figure S2I). In contrast, infection of Cdk2-SN-T160A-Neo^R^ rAAV plus transfection of pX330 CRISPR/Cas9-gCdk2 plasmid resulted in an impressive 44/48 (92%) Neo^R^ colonies containing recombined Cdk2-SN-T160A (Figure 3B). Of the 44 Cdk2-SN-T160A colonies, 41 (93%) were single Cdk2-SN-T160A alleles (Cdk2^+/SN-T160A^) and 3 (7%) were double alleles (Cdk2^SN-T160A/SN-T160A^) (Figure 3C) (Supplementary Figure S2J). We sequenced 4 clones chosen at random and all four contained both the siSN sequence and the T160A mutation (Figure 3D). Similarly to the Cdk2 results, recombination of the Cdk1 gene with Cdk1-SN-T161E-Neo^R^ rAAV plus CRISPR/Cas9-gCdk1 plasmid resulted in 100% recombined clones (40/40) (Supplementary Figure S5). However, we note that unlike Cdk2 where Cdk1 can partially compensate for complete loss of Cdk2 in Cdk2^SN-T160A/SN-T160A^ clones, Cdk2 cannot compensate for Cdk1 loss and we found no double Cdk1^SN-T161E/SN-T161E^ clones. Transfection of either control siCtrl, siWT, siSN or both siWT and siSN into control Cdk2^+/SN^ resulted in allelic-specific depletion of Cdk2 (Figure 3E). Transfection of siWT into Cdk2^+/SN-T160A^ cells resulted in loss of the wild type, active Cdk2 T160 phosphorylated version (Figure 3E), which was accompanied by a characteristic phenotypic cell cycle arrest (Figure 3F), with retention of the inactive Cdk2^SN-T160A^ allele. In contrast, transfection of siSN, resulted in the selective loss of the Cdk2^SN-T160A^ allele with continued expression of the wild type Cdk2 allele and had no effect on cell cycle progression or Cdk2 T160 phosphorylation. Taken together, these observations demonstrate the ability of CRISPR plus rAAV to efficiently recombine a genomic locus and tag it with a selective siRNA sequence that allows for allele-selective phenotypic assays with the gene of interest expressed and regulated under endogenous conditions.

## DISCUSSION

Although recent studies have demonstrated the utility of rAAV to deliver CRISPR–Cas9 components (17–20), none of these studies have used rAAV with CRISPR–Cas9 for targeted gene replacement into the endogenous locus. Here, we designed a highly efficient CRISPR-Cas9 and rAAV targeted gene recombination method to study mammalian gene function by simultaneously introducing phenotypic mutations plus selective siRNA sites into one allele of a gene allowing for expression of a wild type and mutated protein in the same cell. Identifying silent point mutations to generate siSN sequences can be readily performed by transfection of the corresponding expression plasmid and screening siRNA sequences. Combining rAAV with allele-specific siRNAs allows for the selective study of haplo-insufficiency (for siSN) and mutations (for siWT) that are expressed and regulated under endogenous conditions within the same genetic background. Importantly, a recent single-cell RNA-Seq study determined that 76–88% of all human autosomal genes are biallelic (expressed from both alleles) and only 12–24% were mono allelic (21). This suggests that our CRISPR–Cas9 and rAAV approach should be applicable to an overwhelmingly large number of genes in the human genome.

The ease in generating a specific ‘on-target’ DNA double strand break by applying the CRISPR–Cas9 technology has raised multiple questions as to how to identify potential ‘off-target’ events. Currently, two methods find general approval: (i) using published ‘off-target’-prediction tools to identify the top five hits followed by conventional sequencing. Surveyor or T7EI assay (7,22–26). Additionally, (ii) although costly, whole genome deep-sequencing is also applied. These methods, however, favor the identification of sequence-related and not phenotype-based ‘off-target’ effects. Combining CRISPR–Cas9 and rAAV with allele-specific RNAi closes this gap. More specifically, if a phenotypic ‘off-target’ event occurred, the phenotype should be prominent regardless of whether the wild-type or mutated allele is silenced. Hence, our method provides the necessary confidence in the resultant cell line and true siRNA-mediated phenotype for subsequent functional studies of the engineered gene.

Collectively, we believe that our CRISPR plus rAAV approach has broad applicability for studying endogenous gene function that greatly overcomes the problems associated with classical gene rescue approaches.

## SUPPLEMENTARY DATA

Supplementary Data are available at NAR Online.

SUPPLEMENTARY DATA
