# Supplementary material for: Efficient CRISPR-rAAV engineering of endogenous genes to study protein function by allele-specific RNAi
Source: Nucleic Acids Res. 2015 Jan 13;43(7):e45. doi: 10.1093/nar/gku1403 (PMC4402508; doi:10.1093/nar/gku1403)
Supplement: SUPPLEMENTARY DATA [file supp_43_7_e45__index.html]

Efficient CRISPR-rAAV engineering of endogenous genes to study protein function by allele-specific RNAi — SUPPLEMENTARY DATA 

# Efficient CRISPR-rAAV engineering of endogenous genes to study protein function by allele-specific RNAi

## SUPPLEMENTARY DATA

**Files in this Data Supplement:**

- SUPPLEMENTARY DATA
