# Supplementary material for: Efficient CRISPR-rAAV engineering of endogenous genes to study protein function by allele-specific RNAi
Source: Nucleic Acids Res. 2015 Jan 13;43(7):e45. doi: 10.1093/nar/gku1403 (PMC4402508; doi:10.1093/nar/gku1403)
Supplement: SUPPLEMENTARY DATA [file supp_gku1403_nar-03187-met-k-2014-File003.pdf]

<u>Supplemental Figures</u>

Efficient CRISPR-rAAV Engineering of Endogenous Genes to Study Protein Function by Allele-Specific RNAi

Manuel Kaulich[1], Yeon J. Lee[1], Peter Lönn[1], Aaron D. Springer[1], Bryan R. Meade[1] and Steven F. Dowdy[1,*]

[1]Department of Cellular and Molecular Medicine, University of California, San Diego, 9500 Gilman Drive, La Jolla, CA 92093, USA.

* To whom correspondence should be addressed.

Steven F. Dowdy
Tel: (858) 534-7772
Fax: (858) 534-7797
Email: sdowdy@ucsd.edu

Figure S1. Design and validation of Cdk2 siWT and siSN siRNAs. (**A**) Immunoblot showing efficiency of siWT in depleting Cdk2 over time vs. control siCtrl. (**B**) Sequencing of siSN and siWT Cdk2 genomic sequences. Note 7 silent point mutations (highlighted in red) design of siSN. (**C**) Immunofluorescence and (**D**) immunoblot analysis of HEK293T cells, treated as indicated, demonstrating the specificity of siWT and siSN in depleting GFP-Cdk2-WT or GFP-Cdk2-SN, respectively. Note: endogenous Cdk2 protein level is not affected by transfection of siSN (d, lower panel).

Figure S2. Identification of recombined Cdk2-SN and Cdk2-SN-T160A clones. (**A**) Genomic region of Cdk2 highlighting positions of primers used for PCR-based identification of recombined Cdk2-SN-SA-IRES-Neo^R clones. Primer pair P1 and P2 identify faithful recombined clones, whereas pair P3 and P4 distinguish between single and double allele recombination events. (**B**) Genomic PCR analysis using primer pair P1/P2 on clones obtained with control Cdk2-SN AAV infection alone, (**C**) nonspecific CRISPR-gRNA plus Cdk2-SN AAV and (**D**) CRISPR-gCdk2 plus Cdk2-SN AAV. (**E**) Genomic PCR analysis using primer pair P3/P4 for detection of double allele recombination of control Cdk2-SN AAV alone, (**F**) nonspecific CRISPR-gRNA plus Cdk2-SN AAV and (**G**) CRISPR-gCdk2 plus Cdk2-SN AAV. Orange asterisks indicate double allele recombinations. (**H**) Genomic PCR analysis using primer pair P1/P2 on clones obtained with nonspecific CRISPR-gRNA plus Cdk2-SN-T160A AAV. (**I**) Genomic PCR analysis using primer pair P3/P4 for detection of double allele recombination of control Cdk2-SN-T160A AAV alone (clone #7) and nonspecific CRISPR-gRNA plus Cdk2-SN AAV (clone #12) and (**J**) CRISPR-gCdk2 plus Cdk2-SN-T160A AAV.

Figure S3. Cdk2 heterozygosity does not affect the human cell cycle. (**A**) Cdk2 protein level in Cdk2$^{+/+}$ and Cdk2$^{+/SN\text{-}NeoR}$ cells prior to neomycin selection cassette removal by Cre-expression. Numbers indicate Cdk2 band intensity normalized to the Tubulin signal. Note: Cdk2$^{+/SN\text{-}NeoR}$ cells contain 50% less Cdk2 than Cdk2$^{+/+}$ cells. (**B**) Time-lapse video microscopy of Cdk2$^{+/+}$ and Cdk2$^{+/SN\text{-}NeoR}$ cells after serum re-stimulation, following cell-cycle re-entry. (**C**) Size-exclusion chromatography of lysates from asynchronously growing parental Cdk2$^{+/+}$ and AAV-targeted Cdk2$^{+/SN\text{-}NeoR}$ REP1 cells. Note: 50% of Cdk2 protein level is sufficient to complex all cyclin A and E.

Figure S4. Cdk2 protein levels after transfection with siWT, siSN or a combination of both. Bar graph representing quantification of Cdk2 protein levels from Fig. 2e based on three independent experiments. Colour code matches Figure 2f.

Figure S5. Identification of recombined Cdk1-SN-T160E clones. (**A**) Genomic region of Cdk1 highlighting positions of primers used for PCR-based identification of faithfully recombined Cdk1-SN-T161E-SA-IRES-Neo^R clones. Primer pair P1 and P2 identify faithful recombined clones, whereas pair P3 and P4 distinguish between single and double allele recombination events. (**B**) Genomic PCR analysis on clones obtained with CRISPR-gCdk1 plus Cdk1-T161E-

SN rAAV with 40/40 (100%) faithful recombination events Genomic PCR analysis using primer pair P1/P2 on clones obtained with Cdk1-T161E-SN AAV plus CRISPR-gCdk1. Bottom band = wild type allele, top band = T161E-siSN recombined allele.

Kaulich et al., Supplementary Figure 1

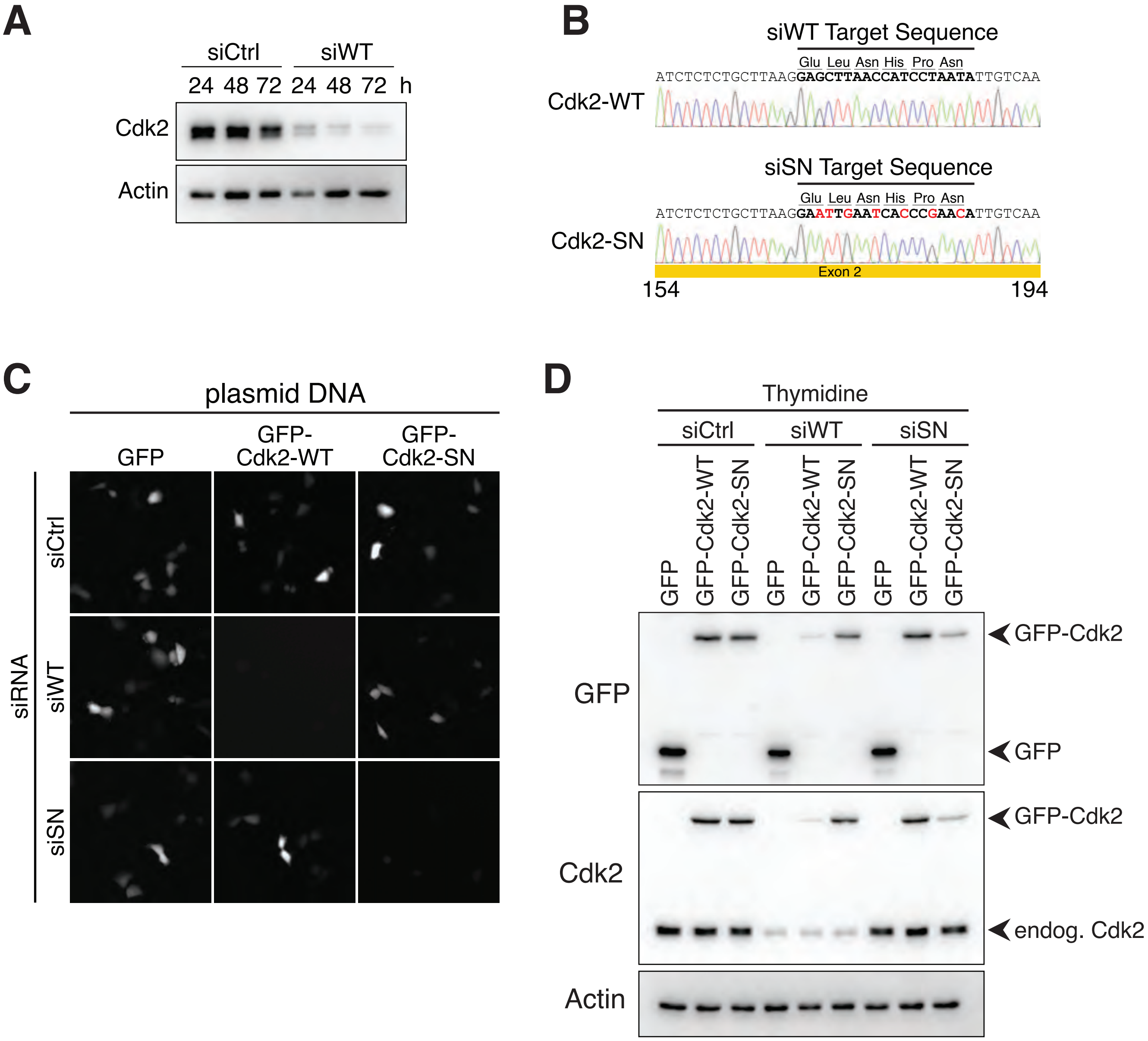

Kaulich et al., Supplementary Figure 2

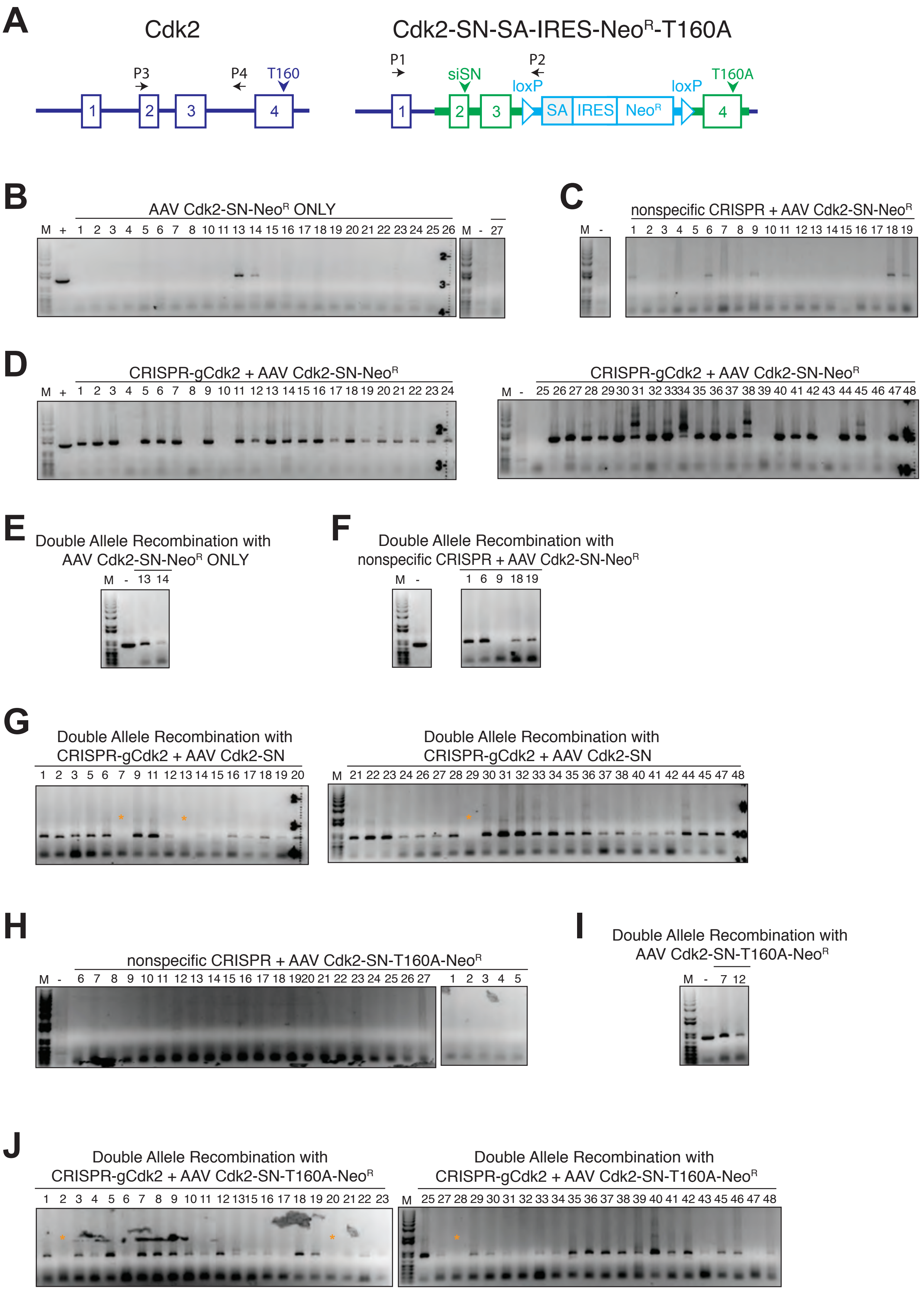

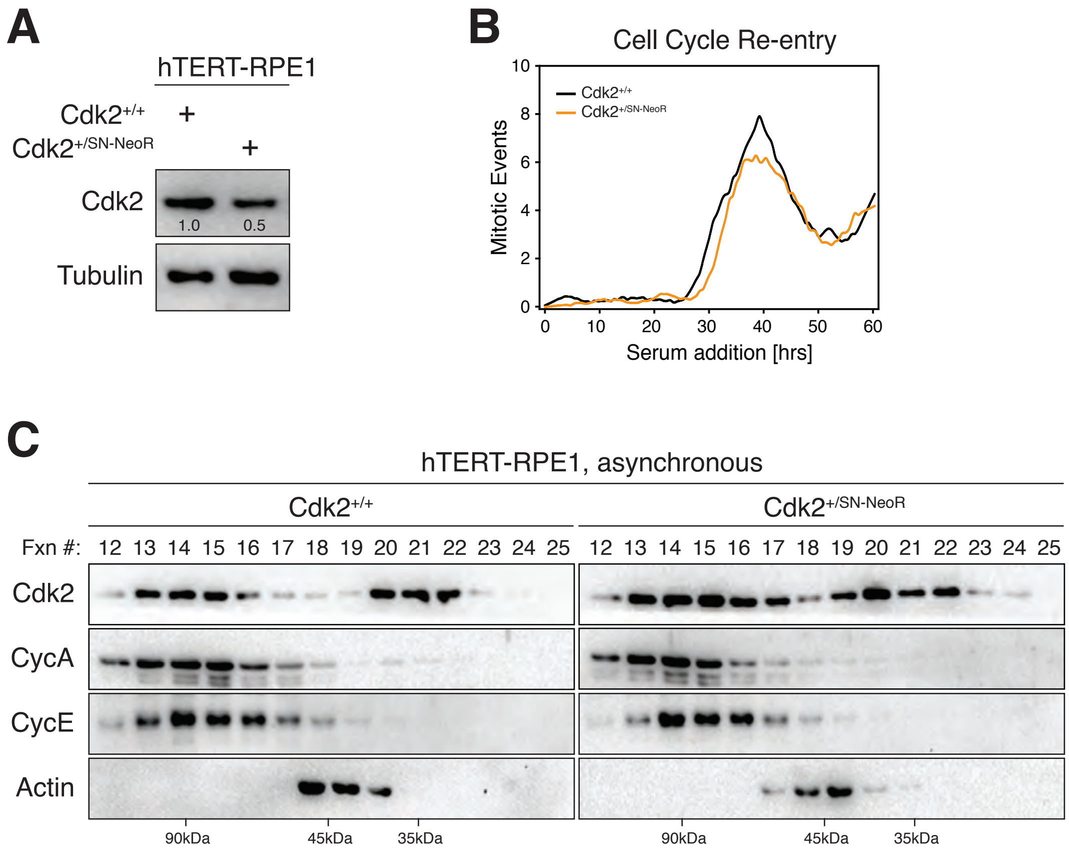
A
hTERT-RPE1
Cdk2+/+
Cdk2+/SN-NeoR
Cdk2
1.0
0.5
Tubulin
B
Cell Cycle Re-entry
Cdk2+/+
Cdk2+/SN-NeoR
Mitotic Events
Serum addition [hrs]
C
hTERT-RPE1, asynchronous
Cdk2+/+
Cdk2+/SN-NeoR
Fxn #: 12 13 14 15 16 17 18 19 20 21 22 23 24 25
Cdk2
CycA
CycE
Actin
90kDa
45kDa
35kDa

Kaulich et al., Supplementary Figure 4

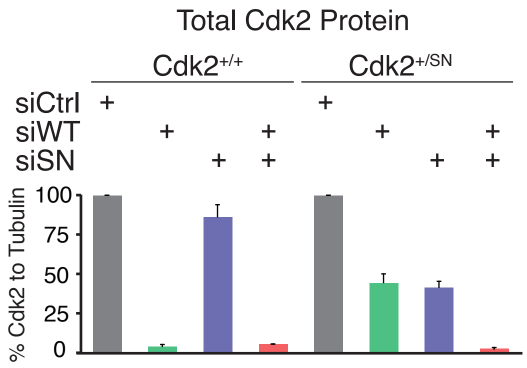

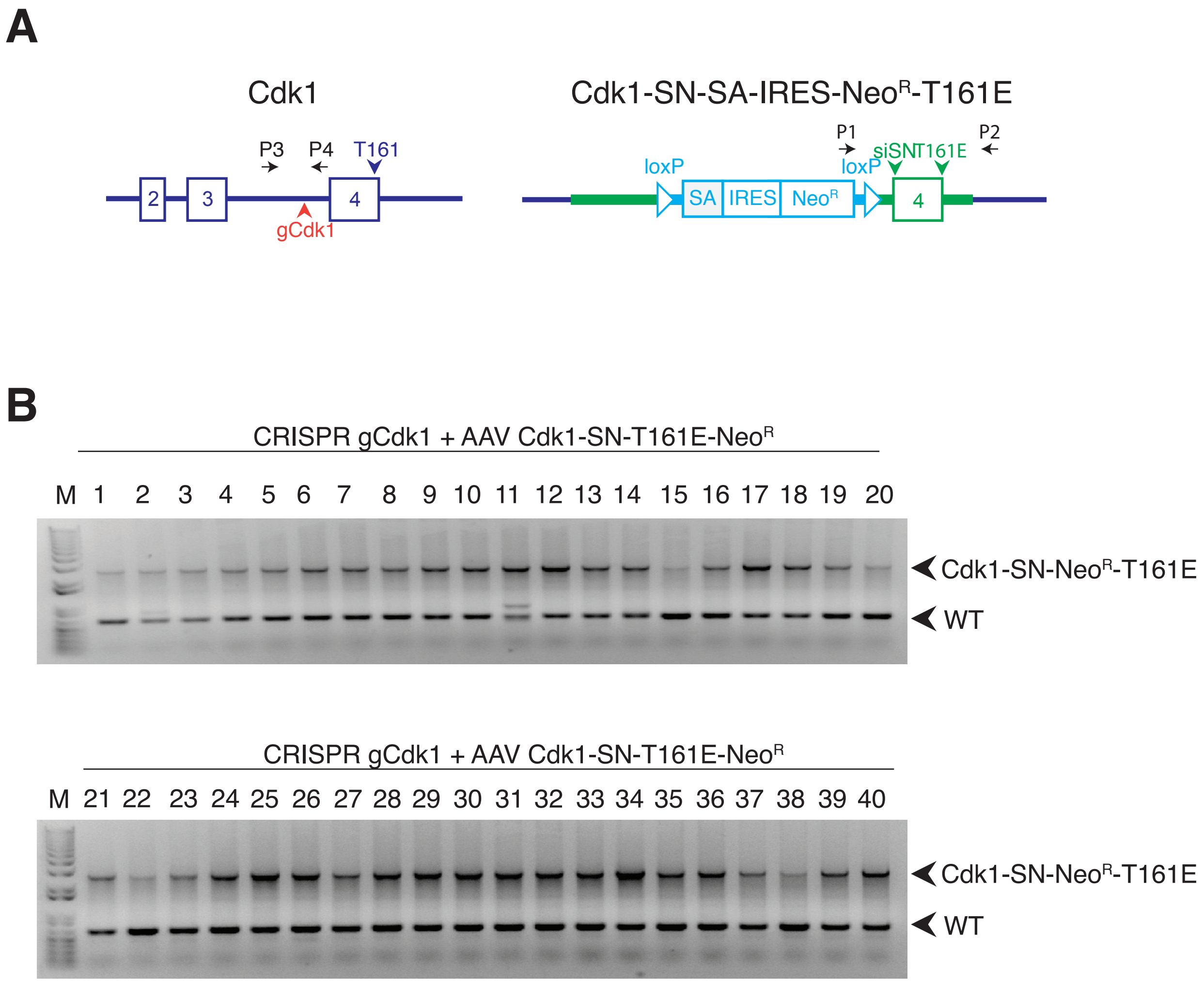
A
Cdk1
Cdk1-SN-SA-IRES-NeoR-T161E
P3
P4
T161
2
3
4
gCdk1
P1
P2
loxP
siSN
T161E
SA
IRES
NeoR
B
CRISPR gCdk1 + AAV Cdk1-SN-T161E-NeoR
M 1 2 3 4 5 6 7 8 9 10 11 12 13 14 15 16 17 18 19 20
Cdk1-SN-NeoR-T161E
WT
CRISPR gCdk1 + AAV Cdk1-SN-T161E-NeoR
M 21 22 23 24 25 26 27 28 29 30 31 32 33 34 35 36 37 38 39 40
Cdk1-SN-NeoR-T161E
WT
